# High-dose folic acid therapy ameliorates gut microbiota dysbiosis in reproductive-aged women with hyperhomocysteinemia

**DOI:** 10.3389/fnut.2026.1832469

**Published:** 2026-08-05

**Authors:** Juanmei Gao, Chao Ye, Yi Jin, Weihua Yang, Jinyi Tong, Hongbo Zhai, Jianmei Xia

**Affiliations:** 1 Department of Obstetrics and Gynecology, Affiliated Hangzhou First People's Hospital, Westlake University School of Medicine, Hangzhou, Zhejiang, China; 2 The Fourth School of Clinical Medicine, Hangzhou First People’s Hospital, Zhejiang Chinese Medical University, Hangzhou, China; 3 Department of Laboratory Medicine, Affiliated Hangzhou First People's Hospital, Westlake University School of Medicine, Hangzhou, Zhejiang, China

**Keywords:** 16S rRNA sequencing, folic acid, gut microbiota, hyperhomocysteinemia, metabolic pathways

## Abstract

**Background:**

Hyperhomocysteinemia (HHCY) in reproductive-aged women is associated with adverse pregnancy outcomes. Although folic acid effectively lowers serum homocysteine (HCY), its relationship with the gut microbiota remains unclear. This study investigated gut microbial alterations associated with HHCY and explored microbiota changes accompanying high-dose folic acid intervention.

**Methods:**

Fecal samples from 38 reproductive-aged women, including 19 patients with HHCY and 19 normal controls, were analyzed using 16S rRNA gene sequencing. Three patients with HHCY receiving high-dose folic acid supplementation at 5 mg/day and multivitamins were additionally monitored longitudinally. Serum HCY concentrations were measured using an enzymatic cycling assay.

**Results:**

No significant differences in alpha diversity were observed between the HHCY and control groups. However, HHCY was associated with an increased abundance of Proteobacteria and several genera, including *Dialister* and *Escherichia-Shigella*, and a decreased abundance of the *[Eubacterium]_eligens_group*. Several differentially abundant taxa were significantly correlated with serum HCY concentrations. Predicted functional analysis indicated alterations in pathways related to amino acid, carbohydrate, cofactor, and vitamin metabolism. In the three longitudinally monitored patients, high-dose folic acid supplementation was accompanied by decreased serum HCY levels and genus-level microbiota changes, with substantial interindividual variability.

**Conclusion:**

HHCY in reproductive-aged women is associated with a specific gut microbial signature rather than a global loss of microbial diversity. The longitudinal observations suggest that high-dose folic acid supplementation may coincide with microbiota alterations; however, the small sample size precludes causal conclusions. Larger, controlled longitudinal studies are required.

## Introduction

Hyperhomocysteinemia (HHCY) in reproductive-aged women is a well-documented risk factor for adverse pregnancy outcomes, including preeclampsia, fetal growth restriction, and neural tube defects (NTDs) (1–3). Clinically, HHCY is classified based on plasma HCY concentrations: 10 to 15 μM cause mild HHCY, 16 to 30 μM moderate HHCY, and >30 μM severe HHCY symptoms (4–6). Homocysteine (HCY), a sulfur-containing amino acid central to one-carbon metabolism, serves as a critical link between metabolic imbalance and reproductive health. Elevated maternal HCY levels not only induce placental vasculopathy but also correlate with congenital heart disease (CHD), particularly when first-trimester concentrations exceeding 8.59 μmol/L confer a 5.22-fold increased risk of CHD (7–9).

Folic acid (FA), an essential B-vitamin that facilitates one-carbon transfer reactions, is clinically proven to lower serum HCY and reduce the incidence of NTDs. This is supported by global public health strategies, such as China’s recommendation of 0.4 mg/day FA supplementation from the preconception period through the 12th gestational week (10–13). However, the observation of suboptimal HCY-lowering responses in some women suggest the existence of uncharacterized regulatory factors, with the gut microbiota emerging as a potential associated component rather than a direct mediator.

The gut microbiota plays a pivotal role in modulating host metabolic health during pregnancy (14). Recent evidence suggests a complex interplay between HHCY and the gut microbiome, where HHCY may exacerbate metabolic disturbances by altering bile acid metabolism (15, 16). While interventions like polysaccharides, prebiotics, and engineered probiotics can regulate the gut microbiota and ameliorate metabolic disturbances (17–19), the specific effects of high-dose FA on the microbiota in HHCY-affected reproductive-aged women have not been addressed. Notably, folate-producing *Lactobacillus* strains have shown promise in alleviating folate deficiency and HHCY in experimental models (20), and maternal folate levels correlate with the abundance of specific microbial taxa in offspring (21, 22). However, direct evidence characterizing microbial signatures associated with HHCY in this specific population remains scarce, as does our understanding of how high-dose FA therapy might coincide with shifts in the gut microbiota.

In this study, we aimed to identify HHCY-associated gut microbial signatures in reproductive-aged women using 16S rRNA gene sequencing. We further evaluated the effects of high-dose FA supplementation (5 mg/day) on serum HCY levels and gut microbiota composition in a small longitudinal cohort of HHCY patients. Given the observational nature of microbiome profiling, we hypothesized that distinct microbial communities are associated with HHCY status and may be altered following FA intervention. Our findings demonstrate a significant association between specific gut dysbiosis and HHCY, and highlight concurrent microbiota alterations following high-dose FA administration. These insights provide a theoretical basis for further exploring targeted microbiota interventions to optimize FA-based strategies for pregnancy-related metabolic disorders.

## Materials and methods

### Clinical characteristics

A total of 19 patients with HHCY and 19 healthy individuals (Normal Control, NC) were recruited for this cross-sectional study at Hangzhou First People’s Hospital between October 2022 and June 2023. Inclusion criteria for HHCY patients were as follows: (1) ≥ 25 but < 35 years old; and (2) confirmed to have high HCY (>10 μmol/L). The inclusion criteria for healthy individuals were as follows: (1) ≥ 25 but < 35 years old and (2) confirmed to have low HCY (<10 μmol/L). All participants in the NC group had normal bowel habits. Both the NC and HHCY groups received 0.4 mg of oral folic acid (Folic Acid Tablets, Jiangsu Lianhuan Pharmaceutical Co., Ltd.) during the perinatal period, which was provided free of charge through the national community distribution program for women of childbearing age. Exclusion criteria for both groups included the use of antibiotics, probiotics, prebiotics, or symbiotics within the preceding 2 months. The study protocol was approved by the Clinical Research Ethics Committee of Hangzhou First People’s Hospital (permission number: IIT-20220627-0101-01).

### Sample collection

Following an overnight fast (≥8 h), morning stool samples were collected from all participants. Each sample was immediately divided into five aliquots of approximately 200 mg, placed in sterile, pre-chilled cryotubes, transported on ice, and stored at −80 °C until DNA extraction.

### Pharmacological treatment and monitoring

Three patients in the HHCY group were monitored longitudinally. After HHCY was confirmed, they were prescribed high-dose folic acid (5 mg/day) (Folic acid tablets, #H20243422, Jinan Yongning Pharmaceutical Co., Ltd.) and a multivitamin supplement (Elevit Pronatal‌ / Vitamin complex tablets, #HJ20140802‌, Bayer Healthcare Co., Ltd.). Serum HCY levels were re-evaluated at regular intervals.

*Case 1 (SY)*: Diagnosed with HHCY at 6 weeks of pregnancy. Following treatment, HCY levels were retested weekly until 10 weeks. Her HCY level decreased to <8 μmol/L within 1 month and remained stable thereafter. An ankylosing spondylitis flare-up was diagnosed during the second trimester.

*Case 2 (WHH)*: Diagnosed with HHCY during a pre-pregnancy check-up. Treatment was initiated, and a follow-up HCY test was conducted half a month later. Her HCY level decreased to <15 μmol/L after 1 month and further to <8 μmol/L after a second month. Conception occurred 1 month after HCY normalization, and the pregnancy was uneventful.

*Case 3 (ZCM)*: Diagnosed with HHCY at 6 weeks of pregnancy. HCY levels were retested weekly until 10 weeks. Her HCY level decreased to <8 μmol/L within 1 month and remained stable. Gestational diabetes mellitus (GDM) was diagnosed during the second trimester.

### Microbial DNA extraction in fecal samples

Microbial DNA Extraction in the Fecal Samples was described in previous article (23). Briefly, the sample was suspended in 790 μL of sterile lysis buffer (4 M guanidine thiocyanate; 10% N- lauroyl sarcosine; 5% N-lauroyl sarcosine-0.1 M phosphate buffer [pH 8.0]) in a 2 mL screw-cap tube containing 1 g glass beads (0.1 mm BioSpec Products, Inc., USA). This mixture was vortexed vigorously and then incubated at 70 °C for 1 h. After incubation by bead beating for 10 min at maximum speed. DNA was extracted by following the manufacturer’s instructions for bacterial DNA extraction using the E.Z.N.A.^®^ Stool DNA Kit (Omega Biotek, Inc., GA), which excepted lysis steps, and stored at −20 °C for further analysis.

### PCR amplification

PCR Amplification was described before (23). Briefly, the primers F1 and R2 (5′-CCTACGGGNGGCWGCAG −3′ and 5′-GACTACHVGGGTATCTAATC-C-3′) corresponding to positions 341 to 805 in the *Escherichia coli* 16S rRNA gene were used to amplify the V3 ~ V4 region of each sample by PCR. PCRs were run in a T100 ™ Thermal Cycler PCR system (Bio-Rad Laboratories, Inc., USA) using the following program: 3 min of denaturation at 95 °C followed by 21 cycles of 0.5 min at 94 °C (denaturation), 0.5 min for annealing at 58 °C and 0.5 min at 72 °C (elongation), with a final extension at 72 °C for 5 min.

### Sequencing

Sequencing was described previously (23). Briefly, the amplicons from different samples were purified by Hieff NGS® DNA Selection Beads (YeasenBiotech Co., Ltd., China). The products were indexed and mixed at equal ratios for sequencing by Shanghai Mobio Biomedical Technology Co., Ltd. using the MiSeq platform (Illumina Inc., USA) in accordance with the manufacturer’s instructions.

### HCY test principle

Binding or dimer homocysteine (oxidized form) is reduced to free homocysteine, and then it is catalyzed by cysteine *β*-synthetase (CBS) to react with serine to form cysteine sulfuric acid. Cysteine is then broken down by cysteine *β*-lyase (CBL) to form homocysteine, pyruvate, and ammonia. Pyruvate is then converted to lactate using lactate dehydrogenase (LDH), where nicotinamide adenine dinucleotide (NADH) is used as a coenzyme. The ratio of NADH to NAD^+^ is directly proportional to the concentration of homocysteine (△ A340 nm).

### Bioinformatics and statistical analysis

Raw data were processed and quality-controlled using USEARCH (v11.0.667) with strict filtering criteria: (i) Sample-specific sequences were extracted by exact index matching (zero mismatches); (ii) Sequences with overlap length < 16 bp were discarded; (iii) Sequences with overlap error rate > 0.1 were removed; (iv) Merged sequences shorter than 400 bp were excluded.

Quality-filtered reads were dereplicated into unique sequences, sorted by descending abundance, and clustered into Operational Taxonomic Units (OTUs) following the UPARSE pipeline (v7.1, http://drive5.com/uparse/). Singleton sequences were omitted, and chimeric sequences were removed prior to clustering. OTUs were defined at 97% sequence similarity and taxonomically annotated against the SILVA SSU138 database using QIIME2 (2020.11).

Alpha diversity indices, including the ACE estimator, Chao1 estimator, Shannon-Wiener index, and Simpson index, were calculated using Mothur v1.42.1. Between-group differences were assessed using the non-parametric Mann–Whitney U test (R v3.6.0, stats package). Beta diversity was evaluated based on Bray–Curtis dissimilarity and weighted/unweighted UniFrac distances in QIIME (v1.9.1). Principal Coordinate Analysis (PCoA) was performed, and statistical significance of group separation was tested by PERMANOVA (999 permutations) with the R package vegan v2.5–7.

To identify differentially abundant taxa among groups, the Linear Discriminant Analysis Effect Size (LEfSe, v1.1) algorithm was employed.^1^ Functional profiles were predicted from 16S rRNA gene sequences using PICRUSt2 v2.4.1.^2^ The association between key bacterial taxa and HCY levels was evaluated using Spearman’s correlation analysis. Statistical models for estimating microbial diversity and microbial community comparison methodology and metrics were performed using the software package (23).

## Results

### Population and clinical characteristics

**Figure 1:**
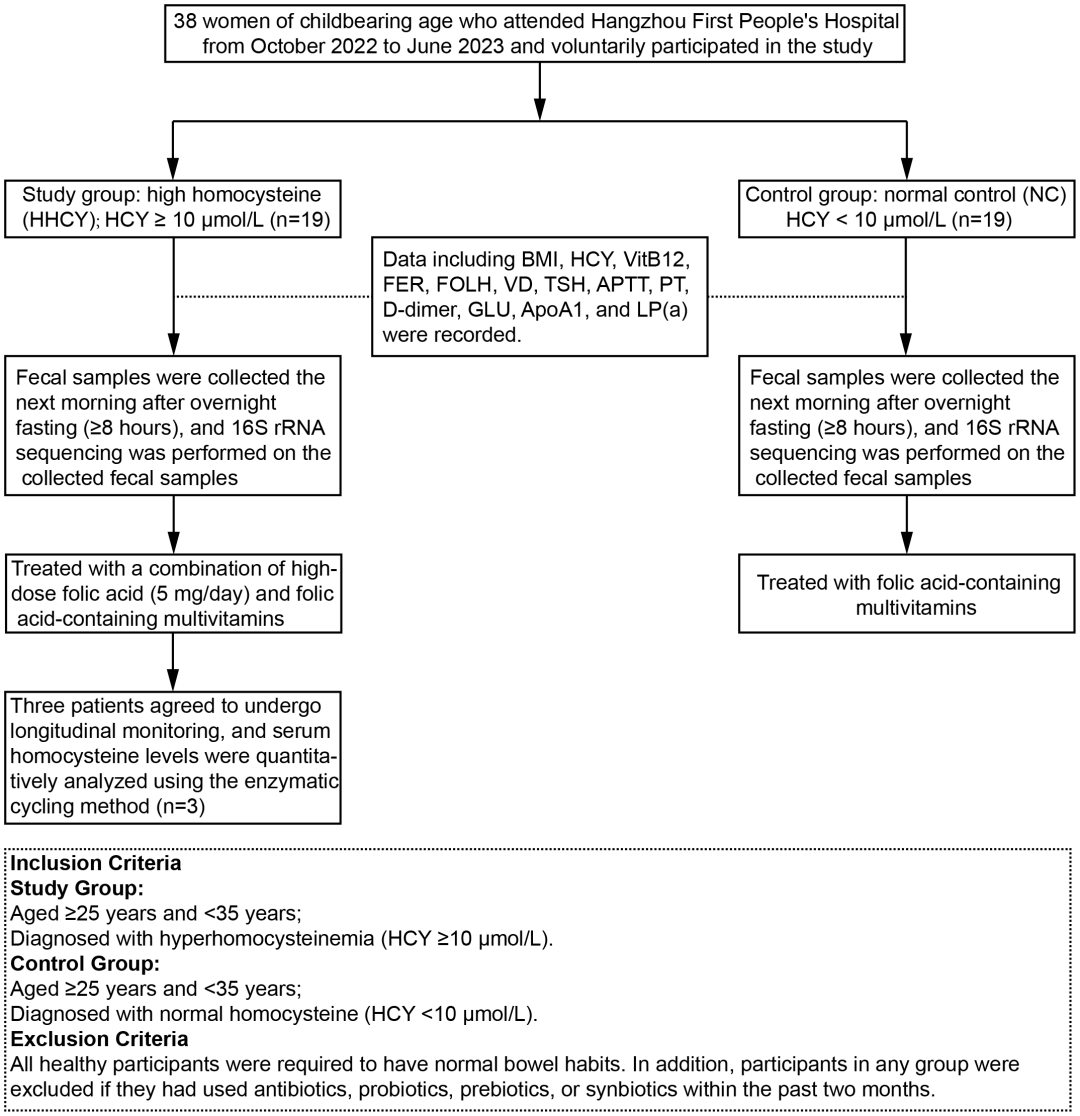
Flow diagram of participants included in this study.

A total of 38 women of reproductive age (19 HHCY patients and 19 NCs) were included in the final analysis. All participants were of Han Chinese ethnicity from the Zhejiang region. A flowchart of the study design is presented in Figure 1 and supplementary information is presented in Supplementary Figure S1. The clinical characteristics of the cohort are summarized in Table 1. As expected, serum HCY levels were significantly higher in the HHCY group compared to the NC group. Additionally, D2 levels were significantly elevated in the HHCY group. There were no significant differences between the groups in terms of age, BMI, or levels of VITB12, FOLW, VD, TSH, and other measured clinical parameters.

**Table 1:** Clinical characteristics of the study cohort.

Feature	NC (*n* = 19)	HHCY (*n* = 19)	*P* value
Age (yr)	31.4 ± 1.0	28.5 ± 1.1	0.0596
Height (cm)	162.0 ± 0.9	163.6 ± 1.0	0.2330
Weight (kg)	56.1 ± 2.1	58.3 ± 2.1	0.4561
BMI	21.3 ± 0.7	21.8 ± 0.9	0.6392
HCY (μmol / L)	8.25 ± 0.19	18.27 ± 2.60	< 0.001
VITB12 (μg/ L)	200.74 ± 17.64	244.95 ± 17.70	0.0886
FER (μg/ L)	84.86 ± 15.78	86.87 ± 15.37	0.9280
FOLW (μg/ L)	9.79 ± 1.11	9.42 ± 1.23	0.8225
VD (ng / mL)	17.26 ± 1.42	15.55 ± 1.02	0.3314
VD2 (ng / mL)	0.70 ± 0.11	0.79 ± 0.11	0.5674
VD3 (ng / mL)	17.69 ± 1.44	15.86 ± 1.00	0.3030
TSH (mIU / L)	2.52 ± 0.34	2.71 ± 0.43	0.7360
APTT (sec)	27.54 ± 0.76	28.80 ± 0.74	0.2427
PT (sec)	11.28 ± 0.15	11.66 ± 0.14	0.0827
D2 (μg/ L FEU)	246.32 ± 20.60	442.11 ± 89.45	<0.05
GLU (mmol / L)	4.72 ± 0.08	4.88 ± 0.10	0.2603
ApoA1 (g / L)	1.62 ± 0.03	1.65 ± 0.06	0.7342
LP (a) (mg / L)	19.57 ± 2.70	28.84 ± 3.89	0.0577

BMI, body mass index; HCY, homocysteine; VITB12, vitamin B12; FER, ferritin; FOLW, folic acid; VD, vitamin D; TSH, thyroid stimulating hormone; APTT, activated partial thromboplastin time; PT, prothrombin time; D2, D-Dimer; GLU, glucose; ApoA1, apolipoprotein A1; LP (a), lipoprotein (a). Data are presented as the mean ± SEM.

### Gut microbial profiles in HHCY and control groups

**Figure 2:**
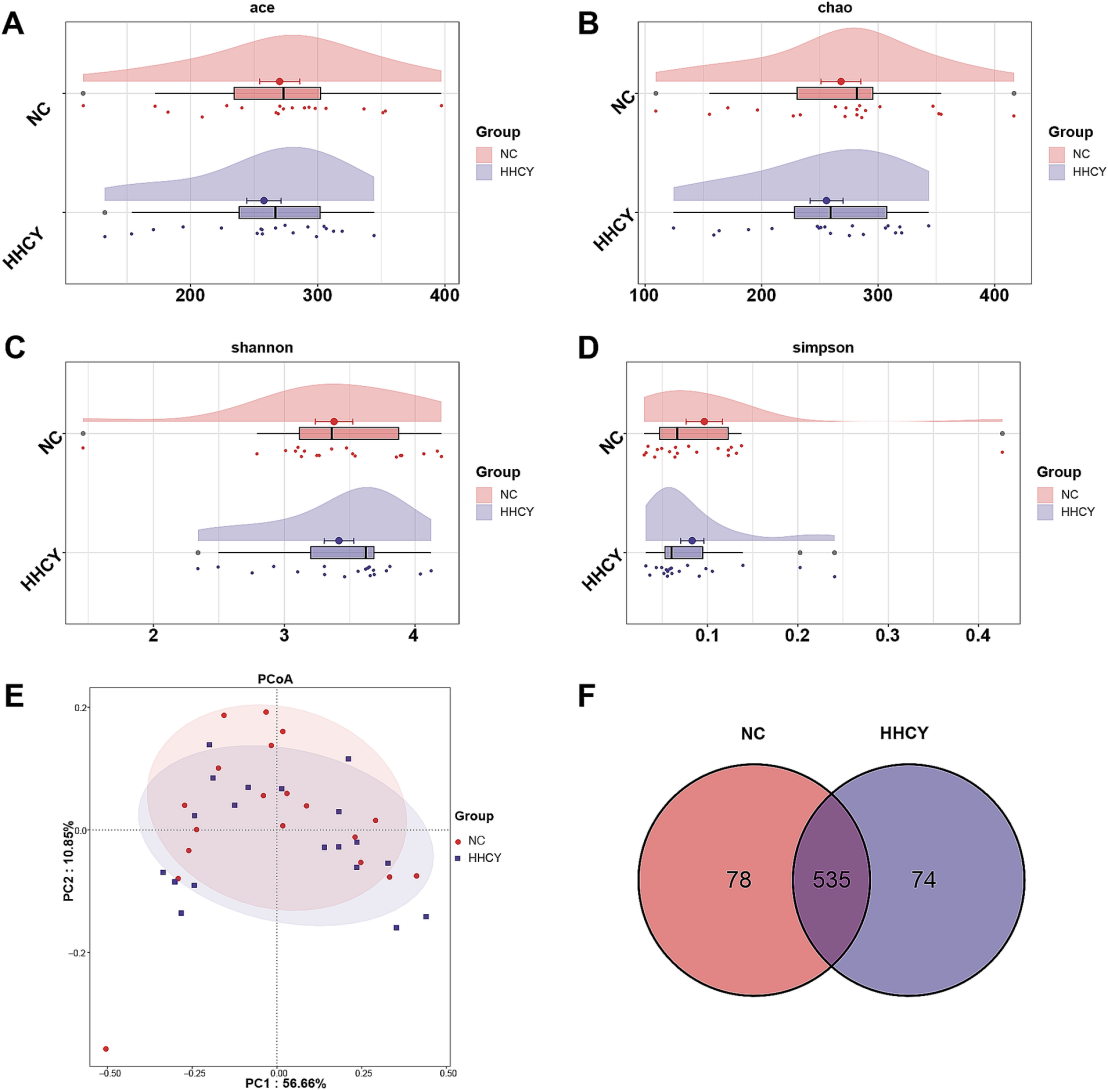
Gut microbiota profile of patients with HHCY and normal controls. **(A)** ACE index; **(B)** Chao 1 index; **(C)** Shannon index; **(D)** Simpson index; **(E)** Comparison of the *β*-diversity of gut microbiota between the HHCY and NC groups; **(F)** Venn diagram of operational taxonomic units (OTUs) in the HHCY and NC groups. NC, Normal control, *n* = 19; HHCY, High homocysteine, *n* = 19.

Analysis of 16S rRNA gene sequencing data revealed no significant differences in *α*-diversity between the HHCY and NC groups, as measured by the ACE, Chao1, Shannon, and Simpson indices (*p* > 0.05 for all; Figures 2A–D). This suggests that the overall richness and evenness of the gut microbial community were preserved in patients with HHCY. Similarly, PCoA based on weighted-UniFrac distances did not show a clear separation between the two groups, indicating a high degree of similarity in the overall microbial community structure (Figure 2E). A Venn diagram showed a substantial overlap of 535 operational taxonomic units (OTUs) between the groups, with only 78 and 74 unique OTUs in the HHCY and NC groups, respectively (Figure 2F). The community composition of the HHCY group was no significantly changed compared to that of the NC group. These results suggest that the gut microbiota of patients with HHCY is generally balanced. Perhaps due to similarities in diet, region, and age, there is no significant difference in the overall gut microbiota between the HHCY group and the NC group.

**Figure 3:**
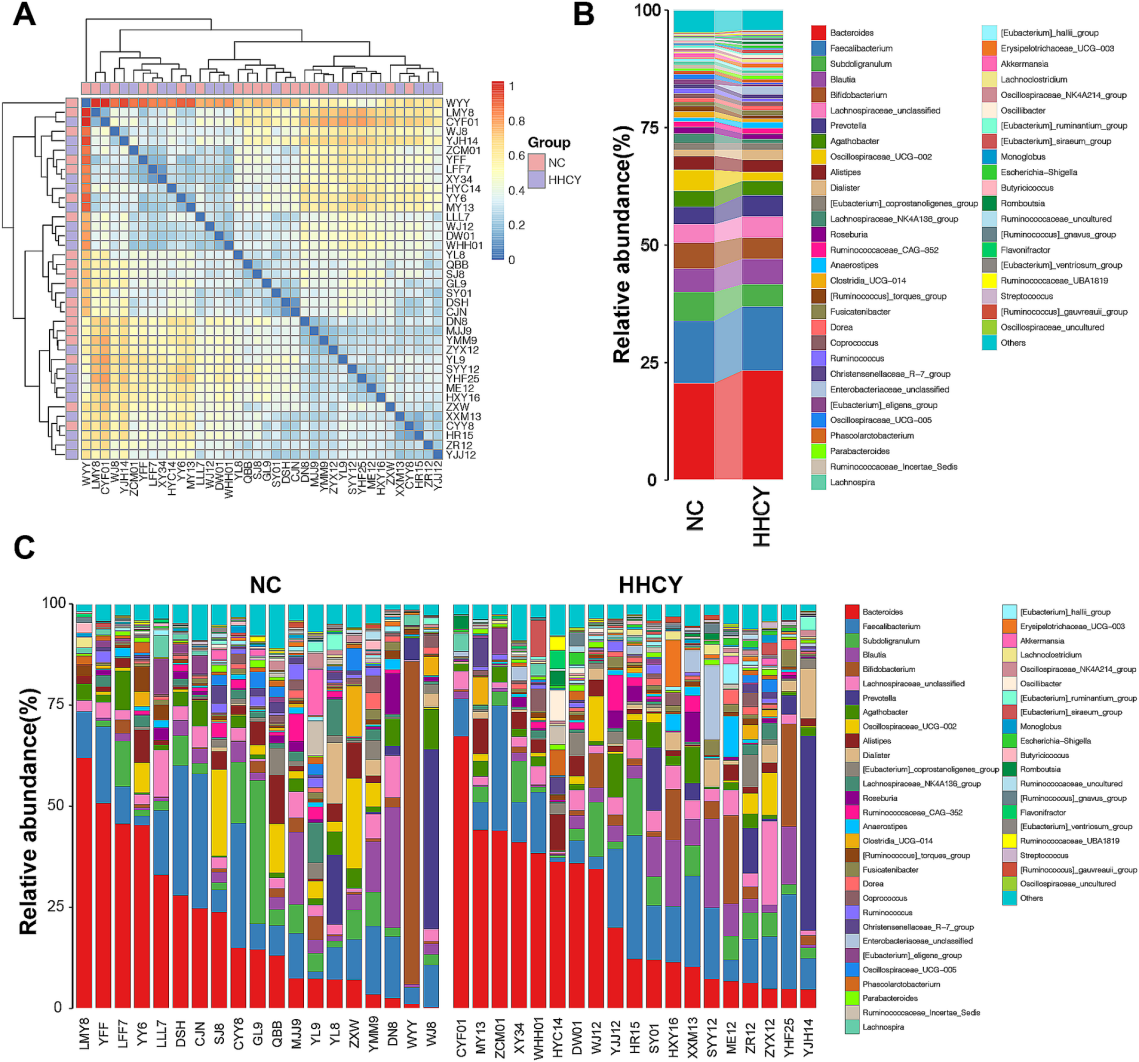
Relative abundance of gut microbiota in patients between HHCY and NC. **(A)** Heatmap analysis based on weighted-UniFrac of beta-diversity in the two groups. **(B)** Column stacking of relative abundance of gut microbiota at genus level in the two groups. **(C)** Component proportion of gut microbiota at genus level in the two groups. NC group: *n* = 19; HHCY group: *n* = 19.

Moreover, the evolutionary differences of the system after abundance weighting were analyzed through heatmap based on weighted-UniFrac (Figure 3A). Through the stacking of bacterial columns of relative abundance and the analysis of bacterial composition ratios, the study found that the bacteria dominant at the genus level mainly included *Bacteroides, Faecalibacterium, Subdoligranulum* and *Blautia*, etc. (Figures 3B,C).

### HHCY-associated dysbiosis in the gut microbiota

**Figure 4:**
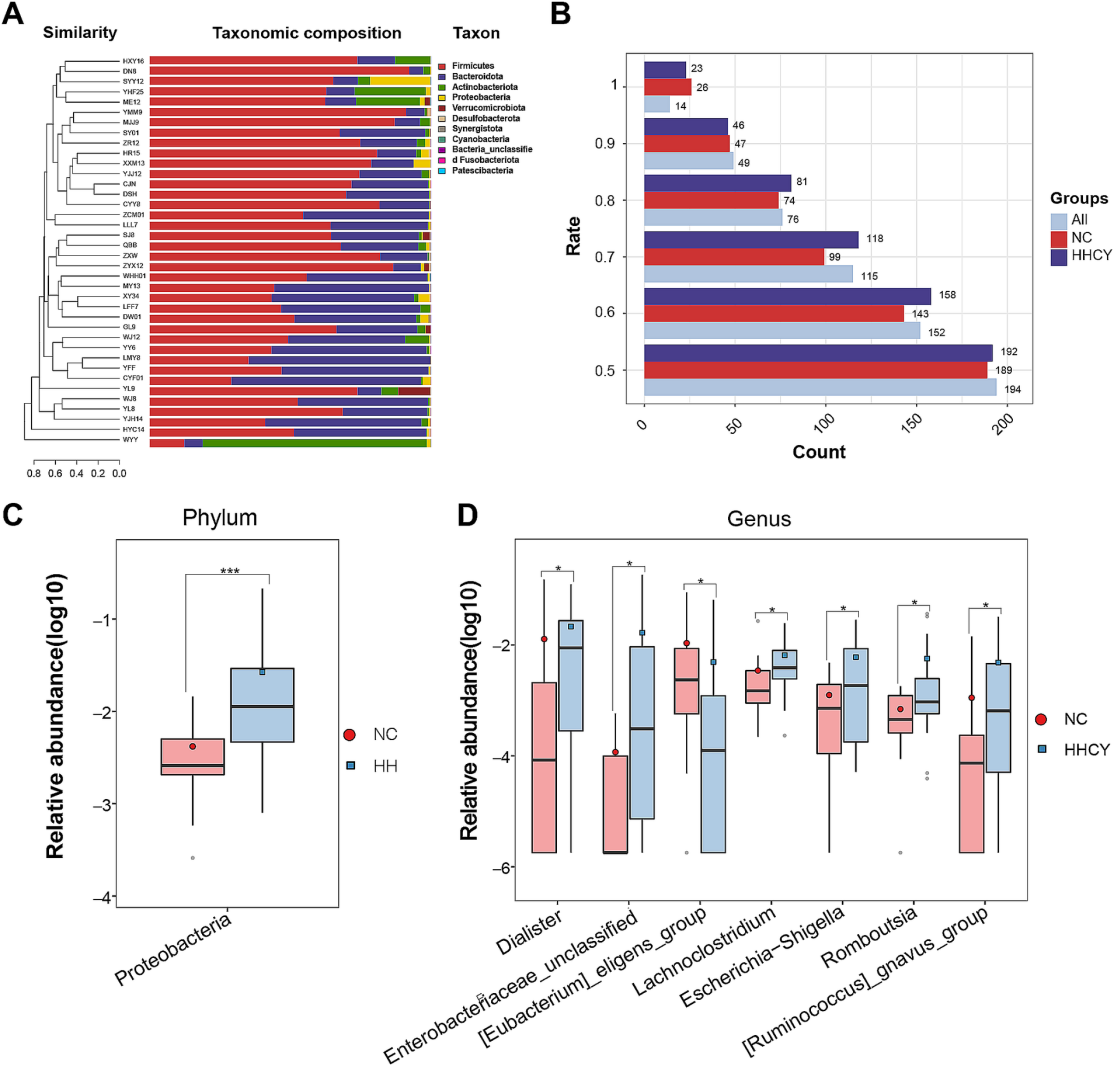
Differentially abundant gut microbiota between HHCY and NC groups. **(A)** Hierarchical cluster bar analysis the phylum-level classification and relative abundance in the two groups. **(B)** The rate of core microbiome in the groups. **(C)** HHCY-related changes in the composition of gut microbiota at the phylum level. **(D)** HHCY-related changes in the composition of gut microbiota at the genus level. *p < 0.05, ** *p* < 0.01, *** *p* < 0.001. NC group: *n* = 19; HHCY group: *n* = 19.

**Figure 5:**
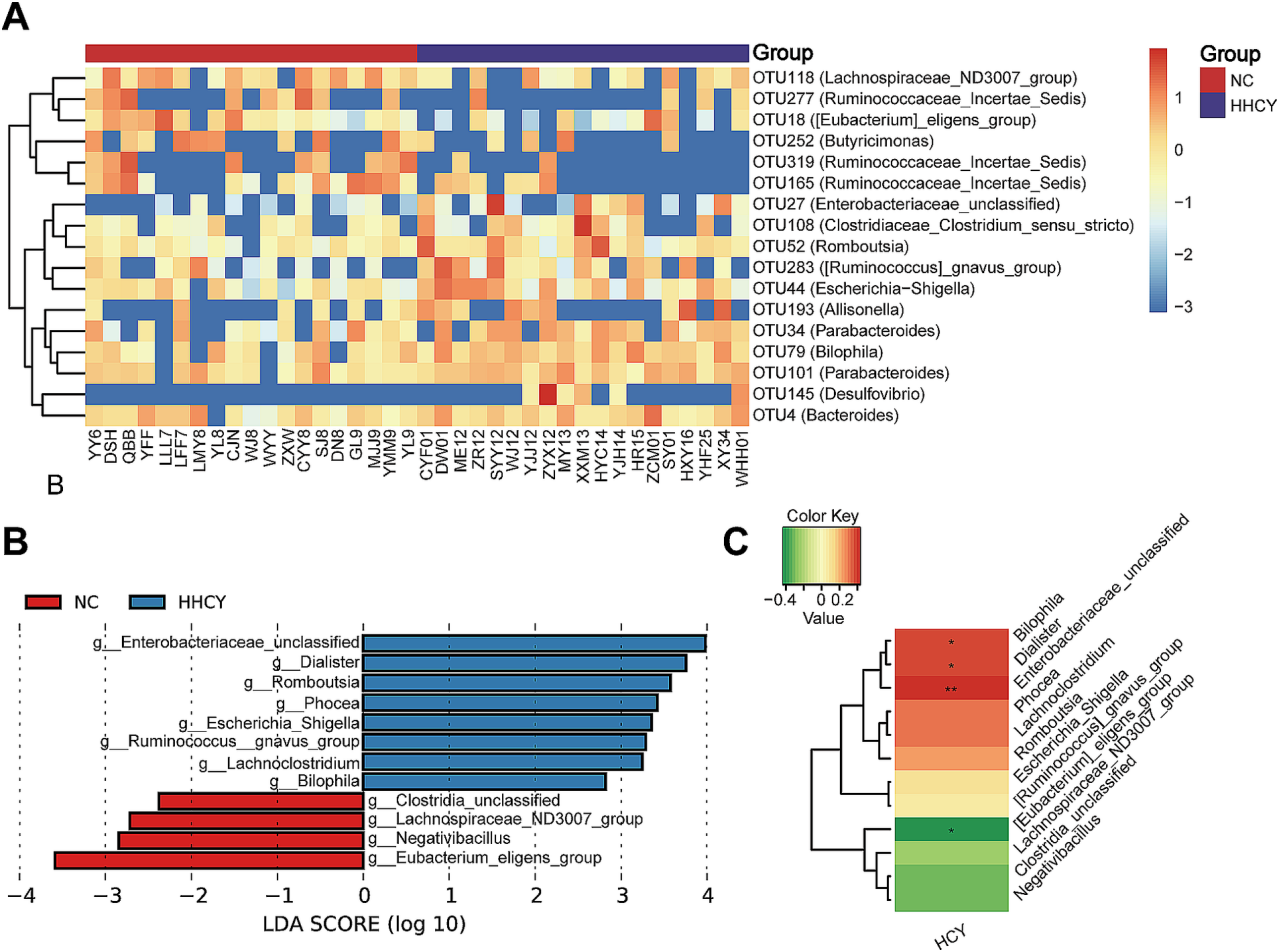
Microbiota changes in patients with HHCY and correlation analysis. **(A)** Heatmap of differentially abundant OTUs identified by Random Forest analysis. **(B)** LEfSe analysis showing significantly different bacterial genera between the two groups. **(C)** Spearman correlation heatmap between differential gut microbiota genera and serum HCY values. *p < 0.05, ** *p* < 0.01, *** *p* < 0.001. NC group: *n* = 1*9; HHCY group: n* = 19.

Despite the similar overall microbial profiles, analysis at finer taxonomic levels revealed significant differences. At the phylum level, the relative abundance of Proteobacteria was significantly higher in the HHCY group compared to the NC group (*p* < 0.05; Figures 4A,C). At the genus level, LEfSe and Mann–Whitney U tests identified several differentially abundant taxa. The HHCY group showed a significant enrichment of *Dialister*, *Lachnoclostridium*, *Escherichia-Shigella*, *Romboutsia*, the *[Ruminococcus]_gnavus_group*, and an unclassified genus from the *Enterobacteriaceae* family. Conversely, the abundance of the *[Eubacterium]_eligens_group* was significantly lower in the HHCY group (all *p* < 0.05; Figures 4D, 5B). These findings suggest that HHCY is associated with a specific dysbiotic signature rather than a global disruption of the microbiota.

### Correlation of microbial taxa with HCY levels and functional pathway analysis

**Figure 6:**
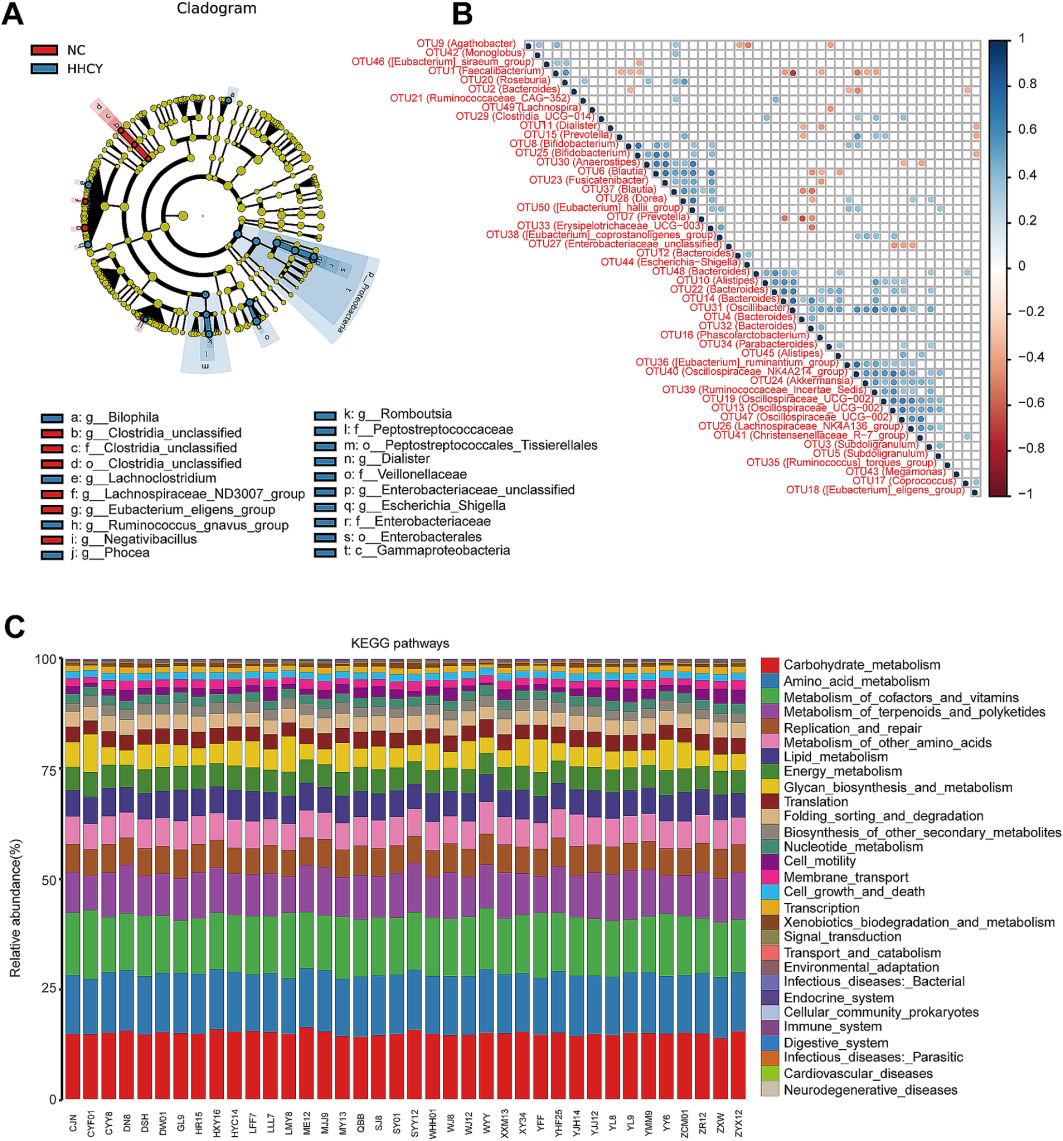
*C*omparative analysis of gut microbiota composition and function. **(A)** Cladogram of gut microbiota by LEfSe analysis between HHCY and NC groups. **(B)** Correlation heatmap of the top 50 OTUs in gut microbiota between HHCY and NC groups. **(C)** KEGG pathway of gut microbiota between HHCY and NC groups.

To determine if the observed dysbiosis was linked to HCY levels, we performed a Spearman correlation analysis. The results demonstrated that the abundances of *Bilophila*, *Dialister*, and the unclassified Enterobacteriaceae genus were significantly and positively correlated with serum HCY concentrations. In contrast, the abundance of the *[Eubacterium]_eligens_group* showed a significant negative correlation with HCY levels (Figure 5C). A random forest analysis further highlighted these OTUs as important discriminators between the groups (Figure 5A). LEfSe cladogram analysis demonstrated distinct phylogenetic divergence of gut microbiota between HHCY and NC groups across multiple taxonomic ranks, confirming a structural microbial dysbiosis in HHCY (Figure 6A). The correlation heatmap of the top 50 OTUs further illustrated a broad, coordinated shift of core bacterial taxa associated with HHCY phenotype, forming clear positive and negative correlation clusters (Figure 6B). KEGG pathway analysis predicted functional alterations in the HHCY gut microbiome. Pathways related to carbohydrate metabolism, amino acid metabolism, metabolism of cofactors and vitamins, and metabolism of terpenoids and polyketides were among the most significantly perturbed, suggesting that the microbial dysbiosis in HHCY may have tangible metabolic consequences (Figures 6A–C).

### High-dose folic acid supplementation modulates HCY and gut microbiota

**Figure 7:**
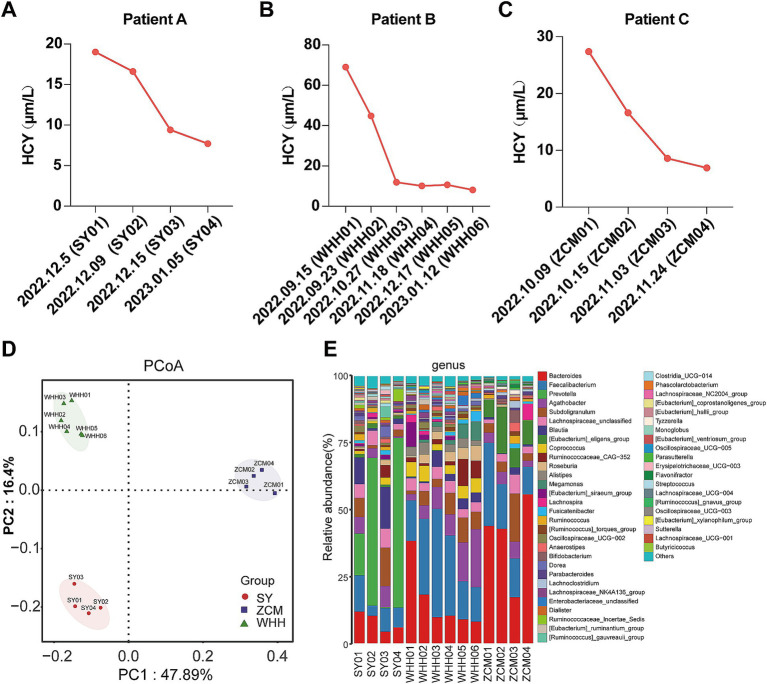
Effects of folic acid supplementation on HCY levels and gut microbiota composition in three patients. **(A–C)** HCY changes in the process of folic acid supplementation in three patients. **(D)** Comparison of the *β*-diversity of gut microbiota in three patients during folic acid supplementation. **(E)** Component proportion of gut microbiota at genus level in three patients during folic acid supplementation.

The longitudinal monitoring of three HHCY patients demonstrated the clinical efficacy of high-dose FA (5 mg/day) supplementation. In all three cases, serum HCY levels progressively declined and normalized within 1 to 2 months of treatment (Figures 7A–C). We also analyzed the dynamic changes in their gut microbiota during the intervention. PCoA analysis based on unweighted-UniFrac distance revealed that while samples from the same individual clustered together over time, there were significant differences between the three patients, highlighting pronounced inter-individual variability in microbiota composition (Figure 7D). At the genus level, dynamic shifts were observed during FA intervention. For instance, a consistent reduction in the relative abundance of *Bilophila* was observed in all three cases, corresponding with the decrease in HCY levels (Supplementary Figure S2). Other genera also showed variable changes over the treatment period (Figure 7E). These findings indicate that high-dose FA may not only mitigates HCY elevation but also actively remodels the gut microbiota profile, linking its therapeutic effects to microbial modulation.

## Discussion

We first performed a retrospective study involving 1,279 obstetric inpatients at Hangzhou First People’s Hospital from January to September 2022, and finally enrolled 53 patients with complete data for analysis of delivery outcomes and pregnancy complications (Supplementary Figure S1). Based on these clinical observations, we further conducted a prospective study focused on HCY, gut microbiota and high-dose FA intervention. This study provides novel evidence linking hyperhomocysteinemia in reproductive-aged women to a distinct gut microbial alterations and offers preliminary observations that high-dose folic acid supplementation coincides with shifts in both HCY levels and gut microbiota composition.

Globally, folic acid (FA) fortification policies vary, with mandatory, voluntary, and no fortification covering 32, 53, and 15% of the population, respectively (24). While mandatory fortification effectively elevates plasma folate and reduces neural tube defects, its additional cardiovascular benefits are largely limited to non-fortified or partially fortified regions (25). Currently, China recommends a targeted periconceptional regimen of low-dose (0.4 mg) FA supplementation, rather than universal fortification programs. However, clinical evidence indicates that HHCY persists even in populations with comprehensive nutritional fortification, highlighting a critical and urgent unmet need. This persistence indicates that HHCY pathogenesis is multifactorial, such as MTHFR genetic polymorphisms and concurrent vitamin B12 deficiency can significantly impair folate metabolism and drive HCY accumulation despite adequate FA intake (26, 27). Consequently, factors beyond simple dietary folate deficiency, particularly host genetics and gut microbial dysbiosis, likely play a crucial role in maintaining HHCY, necessitating microbiota-directed investigations.

A key finding of our study is that HHCY is not associated with a loss of overall microbial diversity but rather with a compositional shift, particularly an expansion of the phylum Proteobacteria. Notably, we observed an enrichment of the genera *Dialister*, *Escherichia-Shigella*, and an unclassified member of the Enterobacteriaceae family, all of which were positively correlated with serum HCY levels. These findings align with prior reports linking *Dialister* to altered folic acid metabolism and *Escherichia-Shigella* to systemic inflammation, suggesting their potential association with HCY accumulation. In contrast, the *[Eubacterium]_eligens_group*, which is generally associated with anti-inflammatory effects and improved glucose metabolism (28), was depleted in the HHCY group, highlighting a potential reduction in beneficial metabolic capacity.

Clinical observations in preconception women demonstrated that folic acid supplementation effectively reduced HCY levels, corroborating meta-analytic evidence that 0.5–5.0 mg/day folic acid lowers serum HCY by 15–40% within 6 weeks (29). Consistent with previous studies, folic acid intervention has shown promising potential in regulating gut microbiota in animal models. For example, FA ameliorated intestinal mucosal damage and altered short-chain fatty acid profiles in hyperuricemic mice (30), and improved dyslipidemia induced by high adiposity in both mice and humans (31). A bidirectional interaction between folate and gut microbiota has been proposed, whereby gut microbiota influences folate bioavailability while folate status modulates microbial structure (32).

However, caution should be exercised regarding high-dose folic acid supplementation, particularly considering the potential for interindividual variability. In a small, exploratory longitudinal subset of three patients, high-dose FA (5 mg/day) rapidly normalized serum HCY levels and coincided with dynamic shifts in the gut microbiota. We noted a consistent reduction in *Bilophila* abundance across all three treated patients (Supplementary Figure S2). *Bilophila* is characterized as a bile-tolerant bacterium, and its proliferation is often strongly associated with animal-based diets rich in fats (33–35). Given the small sample size, the reduction of this taxon following FA treatment serves merely as a preliminary observation. The variability in microbial shifts was also reflected in the clinical trajectories of these three patients, highlighting the complexity of gestational metabolic dysregulation. Furthermore, in three small-scale clinical studies, although short-term high-dose FA combined with multivitamin therapy could also reduce HHCY and regulate the gut microbiota, the corrective intervention in two of these cases was initiated after conception. Furthermore, pre-existing autoimmune diseases and diabetes were activated during pregnancy, and primary conditions such as ankylosing spondylitis were still triggered. This suggests that modifying underlying maternal diseases may require earlier intervention, particularly through pre-pregnancy supplementation.

Furthermore, although FA remains the standard treatment for HHCY, caution is warranted regarding high-dose supplementation. Excessive FA intake carries potential adverse health implications. In animal models, supraphysiological FA doses can induce renal tubular injury and fibrosis (36, 37). In pregnant women, excessive FA supplementation may lead to the accumulation of unmetabolized folic acid (UMFA), especially when interacting with certain medications like antiepileptic drugs, which has been linked to potential fetal neurodevelopmental risks (38) and long-term metabolic alterations in offspring (39). Importantly, FA metabolism is highly dependent on the host’s microbial background, with specific gut microbiota signatures influencing folate utilization (40). To mitigate these risks and provide a balanced nutritional intervention, patients in our study received FA as part of a multivitamin formulation rather than as a standalone high-dose treatment. Integrating personalized evaluations, such as microbial profiling and UMFA monitoring, is essential to optimize the risk–benefit ratio of FA therapy in reproductive-aged women (41, 42).

Although the baseline characteristics were generally comparable between the HHCY and control groups, marginal differences were observed in age and Lp(a), while D-dimer levels showed a significant difference. Aging is associated with shifts in microbial diversity and alterations in the abundance of specific bacterial taxa, whereas dyslipidemia and inflammatory may affect intestinal microbial ecology through metabolic and immune pathways. Therefore, the observed microbial differences between HHCY and control groups may partially reflect the influence of these systemic factors rather than being exclusively attributable to HHCY status. However, given the relatively small sample size, particularly for the longitudinal intervention cohort, statistical adjustment for multiple covariates may reduce model stability and increase the risk of overfitting. Future studies with larger cohorts should incorporate multivariable regression models or propensity score-based approaches to adjust for age, Lp(a), D-dimer, and other relevant clinical variables, thereby determining whether the identified microbial signatures remain independently associated with HHCY.

Importantly, our findings must be interpreted within the context of several major limitations. First, the longitudinal intervention arm included only three patients. Therefore, the dynamic microbiota remodeling observed during FA supplementation is highly exploratory, and we cannot definitively conclude that FA mediated microbiota remodeling directly contributes to improved pregnancy outcomes. Second, our study lacked detailed dietary information. Dietary habits, such as fat, fiber, and animal protein intake, which are among the strongest determinants of gut microbiota composition. The observed shifts in taxa such as *Bilophila*, which is highly responsive to dietary fat, may be influenced by unrecorded dietary variations rather than FA alone. Third, the interventional regimen included multivitamin supplementation alongside folic acid, making it difficult to attribute the observed microbial shifts specifically to FA. Finally, the functional insights provided by KEGG pathway analyses are based on 16S derived predictions rather than direct metagenomic or metabolomic measurements. Consequently, these inferred functional profiles represent hypotheses rather than experimentally validated metabolic or mechanistic alterations.

In summary, our study establishes an observational association between HHCY and specific gut microbial compositional shifts in reproductive-aged women. Our preliminary data suggest that high-dose FA therapy coincides with the remodeling of gut microbiota, providing a rationale for future investigations.

## Conclusion

In conclusion, this study demonstrates that reproductive-aged women with HHCY exhibit a distinct gut microbiota alteration, characterized by an expansion of Proteobacteria (including *Dialister* and *Escherichia-Shigella*) and a depletion of the *[Eubacterium]_eligens_group*. These microbial shifts were significantly correlated with serum HCY concentrations. Furthermore, preliminary observations from an exploratory subset of patients suggest that high-dose folic acid supplementation (5 mg/day) not only reduces serum HCY levels but also coincides with dynamic modifications of the gut microbiota. However, given the limitations regarding sample size and dietary confounding factors, these findings should be interpreted cautiously. Future research utilizing large-scale, well-controlled longitudinal cohorts and direct multi-omics approaches is essential to validate these inferred functional relationships and to explore whether microbiota-directed therapies can optimize the management of pregnancy-related metabolic disorders. Given the small sample size, the reduction of this taxon following FA treatment serves merely as a preliminary observation. The variability in microbial shifts was also reflected in the clinical trajectories of these three patients, highlighting the complexity of gestational metabolic dysregulation.

## Data Availability

The sequencing data have been submitted to the NCBI SRA database under accession number PRJNA1297400. All other data supporting the findings of the study are available in the paper and Supplementary material.

## Footnotes

1
https://github.com/SegataLab/lefse

2
https://github.com/picrust/picrust2/wiki

## Supplementary material

SUPPLEMENTARY FIGURE S1 Flow diagram of total participants screened for the initial retrospective study.

SUPPLEMENTARY FIGURE S2 Line graph showing the change in *Bilophila* abundance corresponding to decreased HCY levels in the three treated cases.
